# Construction of a model predicting the risk of tube feeding intolerance after gastrectomy for gastric cancer based on 225 cases from a single Chinese center

**DOI:** 10.18632/oncotarget.21966

**Published:** 2017-10-23

**Authors:** Wu Xiaoyong, Li Xuzhao, Yu Deliang, Yu Pengfei, Hang Zhenning, Bai Bin, Li zhengyan, Pang Fangning, Wang Shiqi, Zhao Qingchuan

**Affiliations:** ^1^ Division of Digestive Surgery, Xijing Hospital of Digestive Diseases, Fourth Military Medical University, 710032, Xi’an, Shaanxi, China; ^2^ Department of Hepatobiliary Surgery, Shanxi Provincial People's Hospital, 030012, Taiyuan, Shanxi, China

**Keywords:** tube feeding intolerance, gastric cancer, gastrectomy, risk facts, predictive model

## Abstract

Identifying patients at high risk of tube feeding intolerance (TFI) after gastric cancer surgery may prevent the occurrence of TFI; however, a predictive model is lacking. We therefore analyzed the incidence of TFI and its associated risk factors after gastric cancer surgery in 225 gastric cancer patients divided into without-TFI (*n* = 114) and with-TFI (*n* = 111) groups. A total of 49.3% of patients experienced TFI after gastric cancer. Multivariate analysis identified a history of functional constipation (FC), a preoperative American Society of Anesthesiologists (ASA) score of III, a high pain score at 6-hour postoperation, and a high white blood cell (WBC) count on the first day after surgery as independent risk factors for TFI. The area under the curve (AUC) was 0.756, with an optimal cut-off value of 0.5410. In order to identify patients at high risk of TFI after gastric cancer surgery, we constructed a predictive nomogram model based on the selected independent risk factors to indicate the probability of developing TFI. Use of our predictive nomogram model in screening, if a probability > 0.5410, indicated a high-risk patients would with a 70.1% likelihood of developing TFI. These high-risk individuals should take measures to prevent TFI before feeding with enteral nutrition.

## INTRODUTION

The incidence of feeding intolerance (FI) in surgical patients with different diseases fluctuates between 3% and 45.4% [1–3]. FI is an external reflection of gastrointestinal (GI) dysfunction [4]. Varying degrees of GI function injury can lead to GI dysfunction. The clinical characteristic of GI dysfunction includes diarrhea, vomiting, stomach retention and bloatings. Although a meta-analysis has confirmed that postoperative complications and length of hospital stay can be significantly reduced by early postoperative enteral feeding in patients with digestive tract tumors [5]. However, FI is the major reason to resist early enteral feeding is confirmed [6–8].

At present, GI symptoms based on subjective indicators are still used to monitor and assess FI [9].To the best of our knowledge, no predictive model exists to identify patients at high-risk for tube feeding intolerance (TFI) after gastric cancer surgery. Direct or indirect GI injury including tissue trauma, anesthesia patterns, reconstruction of the GI tract and ischemia-reperfusion can cause postoperative GI dysfunction and the degree of GI dysfunction is associated with the type and severity of tissue trauma and the level of systemic or localized inflammatory response [10–12].

In our study, we retrospectively analyzed data from patients with early tube enteral feeding after gastric cancer surgery to determine the incidence of TFI and the risk factors for developing TFI. In addition, we attempted to build a prediction model that could identify a high-risk of TFI in postoperative gastric cancer patients.

## RESULTS

### Incidence

The total incidence of TFI was 49.3% (111/225) in patients after gastric cancer. The daily incidence of TFI from study day 1 (D1) to study day 5 (D5) were 38.67%, 40.10%, 42.22%, 32.22%, and 21.78%, respectively. The most common symptom of TFI was bloating 82.88% (92/111), followed by diarrhea, 10.81% (12/111); (Table 1).

**Table 1:** Basis for diagnosis of TFI

GI Symptom and/or Sign	No. (%) With FI
Large GRV	2 (1.8%)
Nausea or vomiting	2 (1.8%)
Abdominal pain and/or distension	92 (82.88%)
Diarrhea	12 (10.81%)
Nausea/vomiting, Large GRV and abdominal pain/distension	3 (2.7%)

### Baseline characteristics

The baseline characteristics of our gastric cancer patients are shown in Table 2. The mean ages of the patients with and without TFI were 58.08 ± 10.47 years and 58.42 ± 11.61 years, respectively; the mean BMIs were 22.55 ± 3.77 kg/m^2^ and 22.42 ± 3.27 kg/m^2^, respectively; and more than 60% of the total group of patients were male. The mean blood losses were 154.14 ± 117.16 ml and 160.70 ± 14.37 ml and the mean operative times were 215.72 ± 58.28 min and 218.55 ± 58.2 min for the with-TFI group and the without-TFI group, respectively.

**Table 2:** Demographic data and clinical characteristics of the gastric cancer patients

Characteristic	FI (*n* = 111)	Non-FI (114)
Age (years, mean [SD])	58.08 ± 10.47	58.42 ± 11.61
Sex, *n* (%)		
Male	82 (73.87%)	86 (75.44%)
Female	29 (26.13%)	28 (24.56%)
BMI (kg/m^2^, mean [SD])	22.55 ± 3.77	22.42 ± 3.27
Diabetes, *n* (%)		
Yes	5 (4.5%)	12 (10.53%)
No	106(95.495%)	102 (89.47%)
NRS 2002, *n* (%)		
> = 3	47 (42.34%)	55 (48.25%)
< 3	64 (57.66%)	59 (51.75%)
FC history, *n* (%)		
No	45 (40.54%)	19 (16.67%)
Yes	66 (59.46%)	95 (83.33%)
ASA score, *n* (%)		
I	3 (2.7%)	2 (1.75%)
II	86 (77.48%)	103 (90.35%)
III	22 (19.82%)	9 (7.89%)
IV	0	0
Preoperative nutrtion support, *n* (%)		
No	42(37.84%)	27 (23.68%)
Yes	69(62.16%)	87 (76.32%)
Modality, *n* (%)		
Open	49 (44.14%)	50 (43.86%)
MIS (laparoscopy/robot)	62 (55.86%)	64 (56.14%)
Extent of gastrectomy, *n* (%)		
Subtotal	42 (37.84)	46 (40.35%)
Total	69 (62.16%)	68 (59.65%)
Tumor depth, *n* (%)		
T1	34 (30.63%)	25 (21.93%)
T2	16 (14.41%)	10 (8.77%)
T3	19 (17.11%)	19 (16.67%)
T4	42 (37.84%)	60 (52.63%)
pain score at 6-hour postoperation, *n* (%)		
≥ 4	62 (55.86%)	36 (31.58%)
< 4	49 (44.14%)	78 (68.42%)
WBC count on the first day after surgery (10 × 10^9^/L, mean [SD])	16.67 ± 4.67	14.74 ± 4.77
Blood loss (ml, mean [SD])	154.14 ± 117.16	160.70 ± 14.37
Operative time (min, mean [SD])	215.72 ± 58.28	218.55 ± 58.26

### Risk factors for postoperative TFI

The risk factors, especially the independent risk factors, for patients after gastric cancer surgery are shown in Table 3. A preoperative history of functional constipation (FC), a preoperative American Society of Anesthesiologists (ASA) score of III, a high pain score at 6-hour postoperation, the need for preoperative nutrition support and a high WBC count on the first day after surgery were risk factors revealed by univariate analysis; a preoperative history of FC (*P* = 0.000, OR = 3.670, 95% CI: 1.858–7.255), a preoperative ASA score of III (*P* = 0.005, OR = 3.548, 95% CI: 0.533–23.604), a high 6-hour postoperative pain score (*P* = 0.000, OR = 3.324, 95% CI: 1.814–6.089) and a high WBC count on the first day after surgery (*P* = 0.002, OR = 1.104, 95% CI: 1.036–1.176) were revealed by multivariable as independent risk factors for TFI in patients after gastric cancer surgery, compared with other factors the high WBC count on the first day after surgery had a weaker correlation with TFI. The Hosmer-Lemeshow goodness-of-fit test showed the high stability of this logistic model(Prob > χ^2^ = 0.427).

**Table 3:** Risk factors for postoperative TFI after EN

	Univariable		Multivariable	
	OR (95% CI)	*P* value	OR (95% CI)	*P* value
Age	0.997 (0.974–1.021)	0.817		
Sex		0.673		
Male	1			
Female	0.879 (0.484–1.598)			
BMI (kg/m^2^)	1.011 (0.938–1.089)	0.783		
Diabetes		0.097		
No	1			
Yes	0.401 (0.136–1.178)			
NRS 2002		0.308		
< 3	1			
> = 3	0.761 (0.450–1.287)			
**FC history**		**0.000**		**0.000**
**No**	**1**		**1**	
**Yes**	**3.409 (1.831–6.346)**		**3.670 (1.858–7.255)**	
**ASA score**		**0.004**		**0.005**
**I**	**1**		1	
**II**	**0.784 (0.154–3.990)**		0.817 (0.142–4.702)	
**III**	**3.111 (0.531–18.224)**		3.548 (0.533–23.604)	
**Preoperative nutrtion support**		**0.018**		
**No**	**1**			
**Yes**	**0.681 (0.496–0.935)**			
Modality		0.699		
Open	1			
MIS (laparoscopy/robot)	0.900(0.527–1.538)			
Extent of gastrectomy		0.966		
Subtotal	1			
Total	0.989 (0.584–1.674)			
Tumor depth		0.120		
T1	1			
T2	1.176 (0.458–3.023)	0.736		
T3	0.735 (0.324–1.668)	0.462		
T4	0.515 (0.269–0.986)	0.045		
**pain score at 6-hour postoperation**		**0.000**		**0.000**
**< 4**	**1**		**1**	
**≥ 4**	**2.741 (1.591–4.725)**		**3.324 (1.814–6.089)**	
**WBC count on the first day after surgery** **(10 × 10**^9^**/L)**	**1.092 (1.03–1.158)**	**0.003**	**1.104 (1.036–1.176)**	**0.002**
**Blood loss (ml)**	**1.000 (0.998–1.002)**	**0.718**		
**Operative time (min)**	**0.999 (0.995–1.004)**	**0.714**		

### Prediction model for FI after gastric cancer surgery

**Figure 1:**
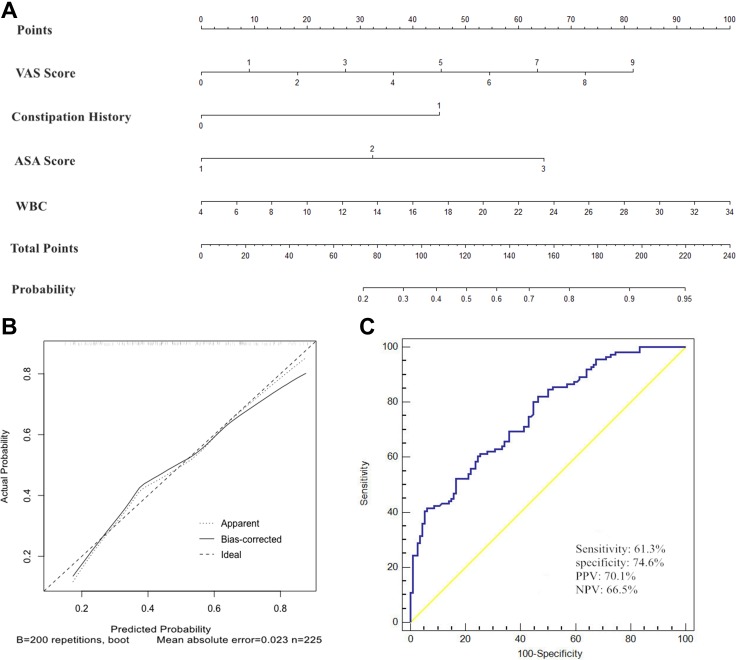
Nomogram, its receiver operating characteristics (ROC) curve and Nomogram calibration plot for predicting postoperative TFI after gastrectomy with lymphadenectomy (**A**) Nomogram from the final multivariable analysis of the binary logistic regression model. (**B**) Nomogram calibration plot. Diagonal reference line indicates the ideal relationship between predicted and actual occurrence of TFI. The mean absolute error was 0.023. (**C**) ROC curve and its diagnostic performance.

To identify patients at high risk of TFI after gastric cancer surgery, we constructed a predictive nomogram model to indicate the probability of developing TFI (Figure 1A). Points were assigned to patients based on their preoperative FC history, preoperative ASA score, 6-hour postoperative pain score and WBC count on the first day after surgery, by finding the appropriate points on the “preoperative FC history”, “preoperative ASA score”, “6-hour postoperative pain score”and “WBC count on the first day after surgery” scales and projecting a vertical line to the “Points” scale at the top of the nomogram. Then, we added these points together and found the corresponding score on the “Total Points” scale. We drew a vertical line and projected it from the “Total Points” scale to the “Probability of TFI” scale to determine the patient’s probability of developing TFI. The actual and predicted probability of the occurrence of TFI after gastric cancer surgery were similar, and the calibration plot showed that the mean absolute error was 0.023 (Figure 1B). Therefore, the model for identifying the patients with a high risk of TFI after gastric cancer surgery was acceptable. Figure 1C suggests that the area under the curve (AUC) for the incidence of TFI is 0.756 according to the ROC curve, and the optimal cutoff for predicting the likelihood of intolerance is 0.5410. The sensitivity, specificity, positive predictive value (PPV) and negative predictive value (NPV) of this model was 61.3%, 74.6%, 70.1%, and 66.5%, respectively.

## DISCUSSION

FI is a common complication and a considerable challenge for early enteral feeding after gastric cancer surgery. A previous study showed that the prevalence of FI among medical and surgical intensive care unit (ICU) patients fluctuated between 2% and 75% [7]. The incidence of FI among surgical patients with different diseases fluctuated between 3% and 45.4% [1–3]. Our study showed that the total incidence of TFI after gastric cancer was 49.3% (111/225), slightly higher than previously reported. Our study only enrolled gastric cancer patients, which may be the reason for the higher incidence of TFI. Our study also showed that the highest daily incidence of TFI occurred from D1 to study day 3 (D3). This coincided with the postoperative acute stress period, during which the body undergoes a series of changes, including nervous system and hormone alterations, resulting in GI absorption injury and dysmotility [3].

FI is a natural external manifestation of GI disorder [4]. A retrospective cohort study showed that enteral feeding can lead to a poor prognosis for patients during the GI disorder period [12]. Therefore, it is important to identify gastric cancer patients at a high risk of TFI at an early stage. To the best of our knowledge, no risk-prediction model for identifying high-risk patients after gastric cancer surgery exists, and this is the first study to create a risk-prediction model for TFI after gastric cancer surgery. The major contribution of this study was the construction and verification of a predictive model by combining four independent risk factors to identify patients at high risk of TFI after gastric cancer surgery.

A previous study showed [3] that age, gender, diabetes, and BMI are correlated with FI. The severity of the disease [4], the stress response to surgical injury (such as operative time, extent and modality), bleeding, and anesthesia [13] are also associated with GI injury, which can result in FI. However, the risk factors for FI still in different diseases remain controversial, especially age [8].

Based on the related literature and clinical experience, we selected 15 potential risk factors to analyze. Our study demonstrated that a preoperative history of FC (*P* = 0.000, OR = 3.670, 95% CI: 1.858–7.255), a preoperative ASA score of III (*P* = 0.005, OR = 3.548, 95% CI: 0.533–23.604), a high 6-hour postoperative pain score (*P* = 0.000, OR = 3.324, 95% CI: 1.814–6.089) and a high WBC count on the first day after surgery (*P* = 0.002, OR = 1.104, 95% CI: 1.036–1.176) are significant predictors of TFI. A preoperative history of FC was the strongest independent predictor of TFI after gastric cancer cancer (OR = 3.670). However, the WBC count on the first postoperative day was a weaker independent risk factor than other factors for TFI patients after gastric cancer (OR = 1.104). These four factors described above were likely identified as independent risk factors, for the following reasons:

First, FC is type of functional bowel disorder, suggesting that such patients already have intestinal functional disorders. In the case of gastric cancer surgery, anesthesia, bleeding and other stress, the body will undergo a series of change including nervous and endocrine system changes, that can cause GI injury [3]. After surgery, the GI functional disorder patients with FC will be aggravated by GI injury. These patients with FC are more likely than others to develop TFI.

Second, the ASA score is proven indicator of the severity of the disease and prognosis [14]. Patients with the higher ASA scores before anesthesia and surgery will have worse organ function. However, the gut is the first organ to be attacked during stress [13] such as tissue trauma, anesthesia, and bleeding, which may aggravate GI functional disorder or failure in patients with a high ASA score. This is accordance with the conclusion that disease severity has been associated with GI dysfunction and failure in patients [4].

Third, postoperative pain can cause an acute response, including stress sympathetic nerve-adrenal medullary reaction enhancement which may result in GI injury. Thus, patients with postoperative pain can develop TFI more easily than patients without pain. Previous studies show that a proper analgesic program after surgery can effectively relieve postoperative pain and promote the recovery of GI function [15, 16].

Finally, WBC count is a sensitive but non-specific marker of acute inflammatory responses [17]. The WBC count is significantly increased during the acute inflammation period and in case ofacute stress after surgical injury, bleeding and anesthesia [18]. The increased WBC count on the first day after surgery may be induced by the postoperative acute stress response. The postoperative WBC count increase is mainly triggered by the severity of tissue trauma surgery and not anesthesia or bleeding [18]. Therefore, the weaker association between TFI and the WBC count on the first day after surgery may be attributed to the introduction of minimally invasive technology.

Risk prediction models based on pre- and post- operative personal information can provide individualized estimates of the risk of TFI after gastric cancer surgery. By identifying the high-risk individuals among the general population, the risk prediction model will help health care professionals enact preventive strategies before enteral feeding. For instance, on the first morning after gastric cancer surgery, we collected data on the four independent risk factors and calculated probability using our nomogram; if a probability > 0.5410, indicated a high-risk patients would with a 70.1% likelihood of developing TFI. Using this nomograph model, we can apply prokinetics early and enact a conservative feeding protocol or use a modified enteral formula to reduce the occurrence of intolerance. A growing body of evidence shows that the use of prokinetics [19] (such as erythromycin and metoclopramide; moderate-quality evidence), modified enteral formula [2, 20–24], feeding protocol (PEP uP protocol) [25], nursing-related factors [26], blood glucose control [27], abdominal massage [28, 29] and acupuncture [30] can effectively decrease the incidence of FI. The overall purpose of this study was to establish and verify a risk prediction model for identifying patients with a high-risk of TFI after gastric cancer surgery. With the identification of these high-risk patients, some prevention measures and appropriate treatments may be able to prevent the TFI, but this needs to be verified with further research.

The other important finding of our study is that our risk-prediction model provides a quantitative, no-cost, easy-to-use risk prediction tool for the prevention of TFI. The four identified independent risk factors can be easily collected on the first morning after gastric cancer surgery. We can then assign points to patients according the nomograph (Figure 1A) and calculate the probability of developing TFI based on the total points for each patient. The use of this model does not require special training, does not increase the surgeon’s workload, and is non-invasive. It has the potential positively impact routine clinical practice in this field, especially in busy gastroenterological surgical units. However, before the predictive nomograph model can be used in clinical practice, a prospective verification study is required.

### Strengths and limitations

These preoperative and postoperative clinical variables can be easily collected on the first morning after gastric cancer surgery. No special training is required to assess the patient’s likelihood of developing TFI and identify high-risk patients quickly, accurately, and conveniently.

There were several limitations to our study: First, our study was only a retrospective study at a single center with a limited sample size. Second, the bloating assessment, VAS score, ASA score and FC are subjective and may result in bias; Third, we cannot confirm whether our results could be replicated in other hospitals or countries; Fourth, we lacked external validation data because of the limited sample size. Therefore, a randomized controlled trial with a large, multicenter sample is needed.

In conclusion, we confirmed that a preoperative history of FC, a preoperative ASA score of III, a high 6-hour postoperative pain score, and a high WBC count on the first day after surgery were independent risk factors for TFI in patients after gastric cancer surgery. We established a no-cost, easy-to-use predictive model for identifying individuals with a high risk of TFI on the first morning after surgery. This model could be used to select individuals who would benefit from appropriate treatments and prevention measures to prevent the occurrence of TFI.

## MATERIALS AND METHODS

### Patient enrollment

**Figure 2:**
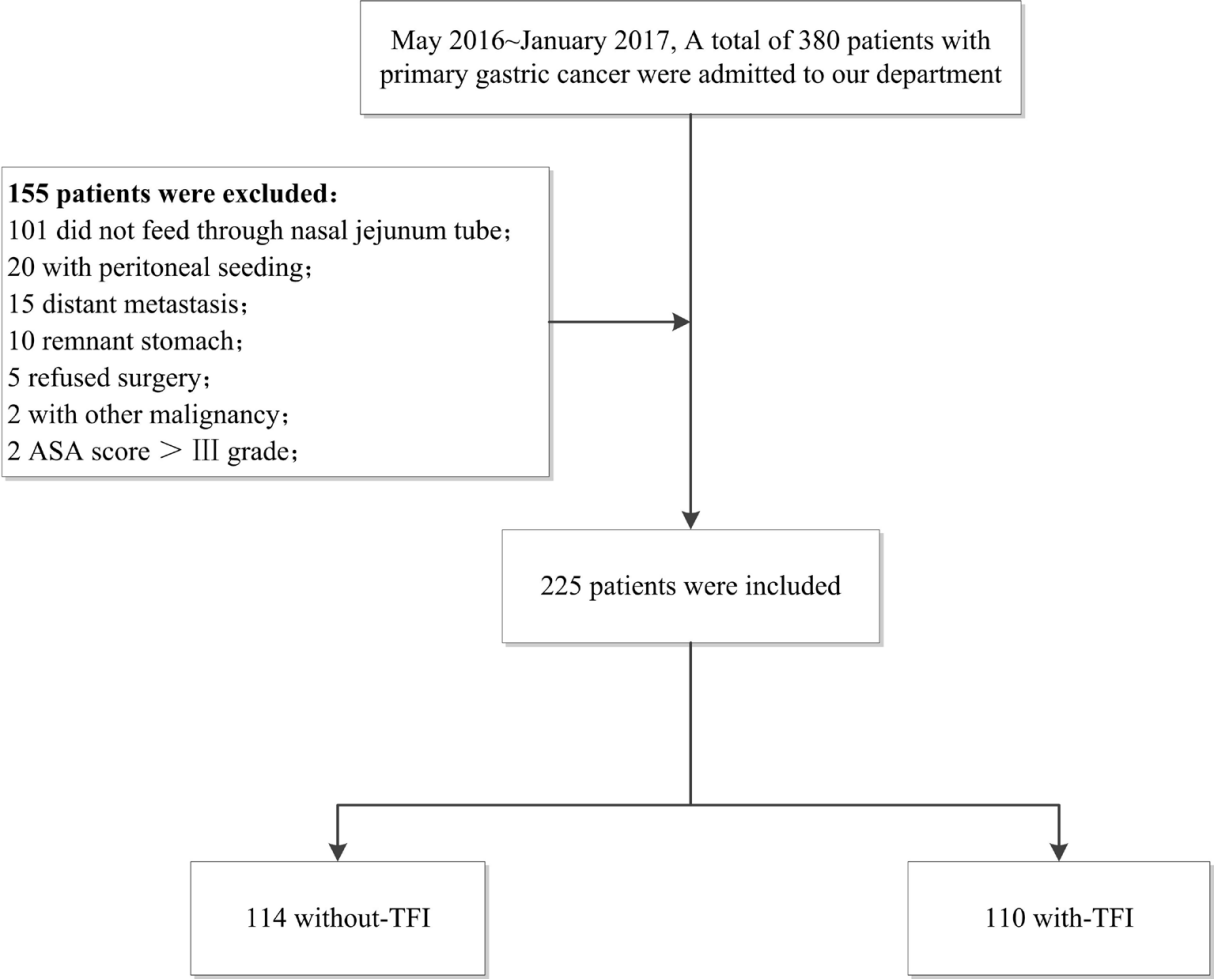
Flowchart of patients’ analysis is shown

A total of 380 patients with primary gastric cancer were admitted to our department (Xijing Hospital of Digestive Diseases affiliated with the Fourth Military Medical University) between May 2016 and January 2017. Patients with residual gastric cancer, or those who underwent palliative surgery due to stage IV tumor (peritoneal implantation or distant metastasis), combined with other malignant neoplasms, refused surgery or had an ASA score > III were excluded. Patients who were not feed through a nasal jejunum tube were also excluded. The remaining 225 patients were divided into two groups according to whether they had FI symptoms (Figure 2). This study was approved by the Ethics Committee of Xijing Hospital, and all patients were signed informed consent before surgery.

### Surgery and evaluations

FC diagnosis was based on the Rome III diagnostic criteria [31]. Pain symptom were recorded at postoperative 6 hours by using a visual analog scale (VAS); this value was defined as 6-hour postoperative pain score. The VAS score ranked from 0 to 10, with 0 representing no pain and 10 representing the greatest tolerable pain. When gastric cancer was located in the lower or middle part of the stomach and it was possible to retain sufficient safe tumor margins, we performed distal gastrectomy. Otherwise, if it was impossible to retain sufficient safe tumor margins or if the gastric cancer was located in the upper part of the stomach, we performed total gastrectomy. We implemented D1+ lymphadenectomy for clinically early gastric cancer or D2 lymphadenectomy for clinically advanced gastric cancer [32].

### TFI symptoms

We defined TFI as any symptoms of GI dysfunction that occurred in patients after gastric cancer surgery during the tube enteral feeding. Among of those GI dysfunction symptoms, we mainly monitor the following: a. Diarrhea: Diarrhea was defined as liquid stools more than 4 times or an estimated stool volume greater than or equal to 200 ml in 24 hours. b. Vomiting: Vomiting was defined as enteral feeding formula liquid shooting out from the mouth. c. Bloating: Bloating was defined as abdominal changes observed during the daily physical examination with tympany and/or the absence of bowel sounds [33]. d. Gastric retention: Gastric retention was defined when the recovered gastric retention amount, which was checked every day was ≥ 473 ml [6].

### Clinical management protocol

To ensure that the tip of the feeding tube reached 20 cm into the output anastomosis, a nasal jejunum feeding tube was placed during surgery. According to the fast-track surgical plan, perioperative gastric cancer patients were all given the same treatment, including preoperative nutrition, preoperative anti-infection treatment, multi-mode analgesia, minimally invasive surgery, avoiding excessive rehydration during operation, early tube enteral feeding and early postoperative mobilization [34].

Tube enteral feeding begins within 6 hours after gastrectomy. The initial dose and speed of tube enteral feeding are based on a feeding protocol (presented in Table 4) and are adjusted in response to symptoms of intolerance. On the day after surgery, recorded as D0, 100 ml of 5 percent glucose injection was applied within 6 hours after surgery. Enteral Nutritional Suspension (TPF-DM; Nutricia Pharmaceutical (Wuxi) Co., Ltd; for non-diabetic patients) or Enteral Nutritional Emulsion (TPF-D; Huarui Pharmaceutical Co., Ltd; for diabetic patients) 500 ml was administered on D1 and then gradually increased to meet the caloric goal. Tube enteral feeding was provided for at least 5 days. The physicians and data collectors who participated in our study were all previously trained and accredited. The daily enteral calorie intake, enteral protein intake, and FIsymptoms were recorded for eash study day. The caloric goal of normal-weight patient was 25 kcal/kg/day; for obese patient, it was 14 kcal/kg/day. For both normal and obese patient, the protein target was 1.0 g/kg/d [35].

**Table 4:** Protocol of the adjustment of feed speed^a^

Study day	Initial feed speed, mL/h	Adjustment of the feed speed
0	10–20	Monitoring the intolerance symptoms every 6 hours;
1	20–40	If the patient is tolerant, raise 20 ml/h from the initial speed
2–5	40–100	If the patient is intolerant; slow down or stop feeding

^a^Study day 0 refers to the surgery day; b.study day 1 refers to after study day 0.

We defined the total incidence of TFI as the occurrence of any symptoms of GI dysfunction during our study period and the daily incidence of TFI as the occurrence of any symptoms of GI dysfunction on any day from D1to D5 after gastric cancer surgery.

### Data collection

In our investigation, we collected the preoperative demographic data and clinical characteristics of the gastric cancer patients, including age, gender, body mass Index (BMI), history of diabetes, 2002 nutrition screening (NRS 2002) score, ASA score, history of FC, modality, extent of gastrectomy, tumor depth, operation time, blood loss, 6-hour postoperative pain score and WBC count on the first day after surgery. We tested all laboratory data in the laboratory of Xijing Hospital in accordance with standard procedures.

### Statistical analyses

Firstly, IBM SPSS for Windows 21.0 software (SPSS, Inc., Chicago, IL, USA) was used to processs the data. The categorical data were reported as numbers with proportions and the quantitative data were reported as means with standard deviation. Risk factors for TFI were identified with odds ratios (ORs) and their 95% confidence intervals (CIs) using a binary logistic regression model. The backward likelihood ratio method was employed to create a final multivariate model and analyze the independent risk factors. Second, the nomogram and its calibration curve were displayed using the package of Regression Modeling Strategies package (package = “rms”; http://CRAN.R-project.org/) in R (version 3.3.3, http://www.R-project.org/), its calibration plot was set at 200 repetitions (B value as 200). Third, a receiver operating characteristics (ROC) curve was constructed using MedCalc Statistical Software (version 17.4; MedCalc Software bvba, Ostend, Belgium; 2017) based on the probability of the final multivariable model and the occurrence of TFI. AUC, and optimal cutoff point (including its sensitivity, specificity, positive predictive value (PPV), and negative predictive value (PPV)) were evaluated using the subject’s ROC curve. A two-sided *p* < 0.05 was considered statistically significant.

## Abbreviations

FI: feeding intolerance
TFI: tube feeding intolerance
ASA: American Society of Anesthesiologists
WBC: white blood cell
GI: gastrointestinal
EN: enteral nutrition
BMI: body mass Index
ORs: odds ratios
CIs: confidence intervals
MIS: minimally invasive surgery
NRS 2002: Nutriton Risk Screening 2002
FC: functional constipation
WBC: white blood cell
VAS: visual analog scale
AUC: area under the curve
ICU: intensive care unit
PPV: positive predictive value
NPV: negative predictive value
ROC: receiver operating characteristics
